# Bridging the leadership gap: agency training and support shapes non-supervisors’ perceptions of their leaders

**DOI:** 10.3389/fpubh.2025.1610400

**Published:** 2025-06-17

**Authors:** Madyson Popalis, Jonathon P. Leider, Avia Mason, Maya Najjar, Moriah Robins, Brian Castrucci

**Affiliations:** ^1^PH WINS, de Beaumont Foundation, Bethesda, MD, United States; ^2^School of Public Health, University of Minnesota, Minneapolis, MN, United States; ^3^Association of State and Territorial Health Officials, Arlington, VA, United States

**Keywords:** public health workforce, training needs assessment, supervisor satisfaction, public health workforce interests and needs survey, PHSSR

## Abstract

**Background:**

Strong leadership is essential for government public health agencies to thrive, as it shapes critical factors like organizational culture, workforce engagement, and job satisfaction. Supervisors serve as key pathways to building and sustaining an effective public health workforce. To strengthen public health leadership, it is important to understand the training and support supervisors currently receive and how this influences the experiences of non-supervisory staff.

**Methods:**

This analysis uses data from PH WINS 2024, a national survey of the state and local public health workforce. Respondents report on demographics, workforce characteristics, and workplace environment. Supervisors reported on agency-provided leadership training and support, while non-supervisors rated their satisfaction with supervisors. A multilevel logistic regression examined how agency and program level supervisor training and support related to non-supervisor satisfaction, adjusting for setting and respondent demographics.

**Results:**

A total of 56,595 employees responded to the survey, with 28% identifying as supervisors. Many supervisors reported gaps in leadership development: 46% did receive initial leadership training, 41% did not receive initial organizational training, 27% do not receive ongoing leadership training, and 31% do not receive ongoing support as a supervisor. The multi-level logit model showed that supervisor satisfaction was more likely at agencies with higher levels of supervisor reported initial training (AOR 1.18, *p* = 0.003) and ongoing training (AOR 1.12, *p* = 0.036). Ongoing support showed the strongest association with supervisor satisfaction (AORs 1.14–1.38, all *p* < 0.005) in agencies where at least half of supervisors reported receiving support.

**Conclusion:**

Key findings from this study indicate that non-supervisory staff report higher satisfaction with their supervisors in agencies where a greater proportion of supervisors received leadership training and have ongoing support. Developing leadership is a practical and powerful way to strengthen the government public health workforce. Prioritizing supervisor training and support as a foundational workforce strategy is key for improving workplace satisfaction. Strengthening public health leadership begins with creating consistent, well-resourced systems for preparing and supporting supervisors.

## 1 Introduction

Strong leadership within state and local health departments has become more essential than ever in guiding the US public health workforce through ongoing challenges. Research across sectors consistently demonstrates effective leadership positively influences organizational culture and enhances employee retention (1, 2). However, leadership is often misunderstood as an innate trait rather than a skill that can be developed through targeted training. As a result, many individuals in supervisory roles receive little to no formal training; this is especially true in public health, where technicians often have management as the sole path of advancement available to them (3). Instead, technical experts are frequently promoted based on their proficiency in a specific subject area, with the added responsibility of directing, guiding, coordinating, and evaluating the performance of their teams. Without formal supervisory training, these professionals often lack the necessary skill to adequately support their teams and have varying levels of understanding of what effective supervision entails (3, 4).

Evidence indicates that leadership skills can be cultivated and enhanced through evidence-based training (5, 6). As the demand for public health leaders who can effectively integrate leadership theory with practice continues to grow, it is imperative that these leaders are equipped not only with strategic decision-making capabilities, but also with the ability to be empathetic people leaders, attuned to the needs of their teams —cultivating a sense of belonging and fostering a supportive work environment. Effective supervision is multi-dimensional, with far-reaching effects across various levels of the organization.

The quality of supervision has been identified as a key factor influencing employee satisfaction, organizational commitment, and intent to remain within an organization. Employees who perceive their supervisors as supportive are more likely to report greater job satisfaction, higher levels of engagement, and a stronger sense of inclusion in the workplace (7, 8). These associations underscore the role of supervisory support in shaping employee experience and offer important context for understanding broader patterns in workforce stability. In the context of public health, where staff are often navigating high workloads and limited resources, supportive supervision may serve as a protective factor against burnout and turnover (9).

Supervisors influence both operational workflows and the interpersonal and developmental climate of their teams. When equipped with strong people-management skills, they are better positioned to foster trust, recognize employee contributions, and promote psychologically safe work environments. Such conditions are associated with improved organizational culture and higher retention rates (10–12). These findings suggest that investment in supervisory training represents a strategic lever for strengthening the long-term capacity and resilience of the governmental public health workforce. Despite the recognized importance of effective supervision, little is known about the extent to which supervisors in government public health are systematically trained or supported in their roles. This analysis seeks to characterize the supervisory government public health workforce and determine the relationship between agency training and support for supervisors and non-supervisor’s perceptions of their supervisors.

## 2 Methods

To examine the relationship between supervisor training and support and non-supervisor satisfaction, a multilevel workforce study was conducted across US health departments in 2024. Data come from the Public Health Workforce Interest and Needs Survey (PH WINS), a nationally-representative survey of state and local government public health employees in the United States conducted in partnership between the de Beaumont Foundation and the Association of State and Territorial Health Officials (ASTHO). Participation for PH WINS first started at the agency level; state and local health officials provided permission for their agency to participate and provided a staff list of all employees at the agency. Participating agencies were then surveyed using a census approach, in which every employee was invited to take the survey. The survey was distributed via email using Qualtrics, a web-based survey platform, and was open between September 9, 2024 and January 17, 2025. The survey was sent to 159,627 employees and 56,595 individuals responded from employees in 48 state health agencies and approximately 1,200 local health departments. After accounting for staff that left their agencies or otherwise had bad contact information, 37% of invitees responded. The requirement of ethical approval for PH WINS 2024 was waived by the WCG Institutional Review Board (Western-Copernicus Group IRB) for the studies involving humans because the research only includes interactions involving educational tests, survey procedures, interview procedures, or observations of public behavior; and there are adequate provisions to protect the privacy of subjects and to maintain the confidentiality of data. The study was conducted in accordance with the local legislation and institutional requirements. The participants provided their written informed consent to participate in this study.

Respondents self-reported individual characteristics including gender, race/ethnicity, age, educational attainment, supervisory status, tenure in public health management, years of supervisory experience prior to their current position, and primary program area. Respondents who identified themselves as supervisors, managers, or executives (hereinafter referred to as supervisors) answered an additional set of questions specific to the experiences of employees in supervisory roles. Supervisors rated four statements related to their agency’s practices on a 4-point Likert scale which were subsequently collapsed into negative (“disagree” or “strongly disagree”) and positive (“agree” or “strongly agree”) response categories.

Similarly, non-supervisors responded using the same 4-point Likert scale for the following statements: “I am satisfied with my supervisor” and “My supervisor is a skilled people manager,” which were collapsed into the same binary categories. Non-supervisors also reported their intention to leave their job within the next year and the reasons why they are considering leaving their job. Balanced repeated replication weights were applied to all analyses to account for the complex design and adjust for non-response. An inferential analysis was conducted wherein staff perceptions around their supervisor’s skill at people management was by agency and program area. A logit model was built, with dichotomized supervisor satisfaction as the dependent variable and setting, respondent demographic, and supervisor support sentiment by agency and program as the independent variables. Supervisor support sentiment was calculated using the four supervisor support questions, averaged by agency and program among supervisors. Instances where there were no supervisors in an agency and program were excluded from analysis. Fit statistics were calculated during model selection. Data were stored, managed, and analyzed using Stata (Stata 17, StataCorp. LLC College Station, TX).

## 3 Results

Among all PH WINS respondents, 28% self-reported as a supervisor or higher, indicating that they are responsible for other employees in some capacity. Most supervisors were female (77%), white (67%), over 40 years of age (72%), and held a bachelor’s degree or higher (83%) (Table 1). Experience levels varied, but 38% of supervisors reported 0–5 years of public health management experience and 46% reported 0–5 years of supervisory experience before their current position, highlighting a workforce that is relatively new to leadership.

**Table 1:** Supervisor demographics and workforce characteristics.

	Percent	95% CI
Gender		
Male	22	[21, 23]
Female	77	[76, 78]
All Other	1	[1, 1]
Race and ethnicity		
American Indian or Alaska Native	0	[0, 1]
Asian	6	[5, 6]
Black or African American	14	[13, 15]
Hispanic or Latino	9	[8, 10]
Middle Eastern or North African	0	[0, 1]
Native Hawaiian or Pacific Islander	0	[0, 1]
White	67	[66, 68]
Multiracial and/or Multiethnic	3	[3, 3]
Age in years		
<30	5	[4, 5]
31–40	23	[22, 24]
41–50	32	[31, 33]
51–60	28	[27, 29]
61+	12	[11, 13]
Highest degree attained		
No college degree	8	[7, 9]
Associates	9	[8, 9]
Bachelors	34	[33, 35]
Masters	40	[39, 41]
Doctoral	9	[8, 10]
Supervisory status		
Supervisor	59	[58, 60]
Manager	29	[28, 30]
Executive	11	[11, 12]
Tenure in public health management		
0–5 years	38	[37, 40]
6–10 years	25	[23, 26]
11–15 years	14	[13, 15]
16–20 years	11	[10, 12]
21 or above	12	[11, 13]
Years of supervisory experience prior to current role		
0–5 years	46	[44, 47]
6–10 years	24	[23, 25]
11–15 years	13	[12, 14]
16–20 years	9	[9, 10]
21 or above	9	[8, 9]

Supervisors reported an absence of leadership training. Nearly half of supervisors indicated that they were not provided with leadership training (46%) or organizational training (41%) upon entry to their current supervisory position (Table 2). Additionally, slightly more than a quarter (27%) reported that they do not receive ongoing leadership training opportunities and 31% reported that they are not provided with ongoing support as a supervisor. Among non-supervisors, 14% expressed dissatisfaction with their supervisors and 18% reported that their supervisors lacked effective people management skills (Table 3). Of non-supervisors who indicated their intent to leave their job in the next year, 23% cited dissatisfaction with their supervisor as a contributing factor to their decision.

**Table 2:** Supervisor training and organizational support.

	Percent	95% CI
My agency provided leadership training when I started		
Strongly disagree	14	[13, 15]
Disagree	32	[30, 33]
Agree	39	[38, 40]
Strongly agree	15	[15, 16]
My agency provided organizational training when I started		
Strongly disagree	11	[10, 11]
Disagree	30	[29, 31]
Agree	46	[44, 47]
Strongly agree	14	[13, 15]
My agency provides ongoing leadership training		
Strongly disagree	6	[5, 7]
Disagree	21	[20, 22]
Agree	54	[53, 55]
Strongly agree	20	[19, 20]
My agency provides ongoing support for supervisors		
Strongly disagree	7	[6, 8]
Disagree	24	[23, 25]
Agree	50	[49, 51]
Strongly agree	18	[18, 19]

**Table 3:** Non-supervisors’ perceptions of their supervisors.

	Percent	95% CI
I am satisfied with my supervisor		
Strongly disagree	4	[3, 4]
Disagree	10	[9, 10]
Agree	40	[39, 41]
Strongly agree	46	[46, 47]
My supervisor is a skilled people manager		
Strongly disagree	5	[5, 6]
Disagree	13	[13, 14]
Agree	41	[40, 41]
Strongly agree	41	[40, 41]

**Figure 1:**
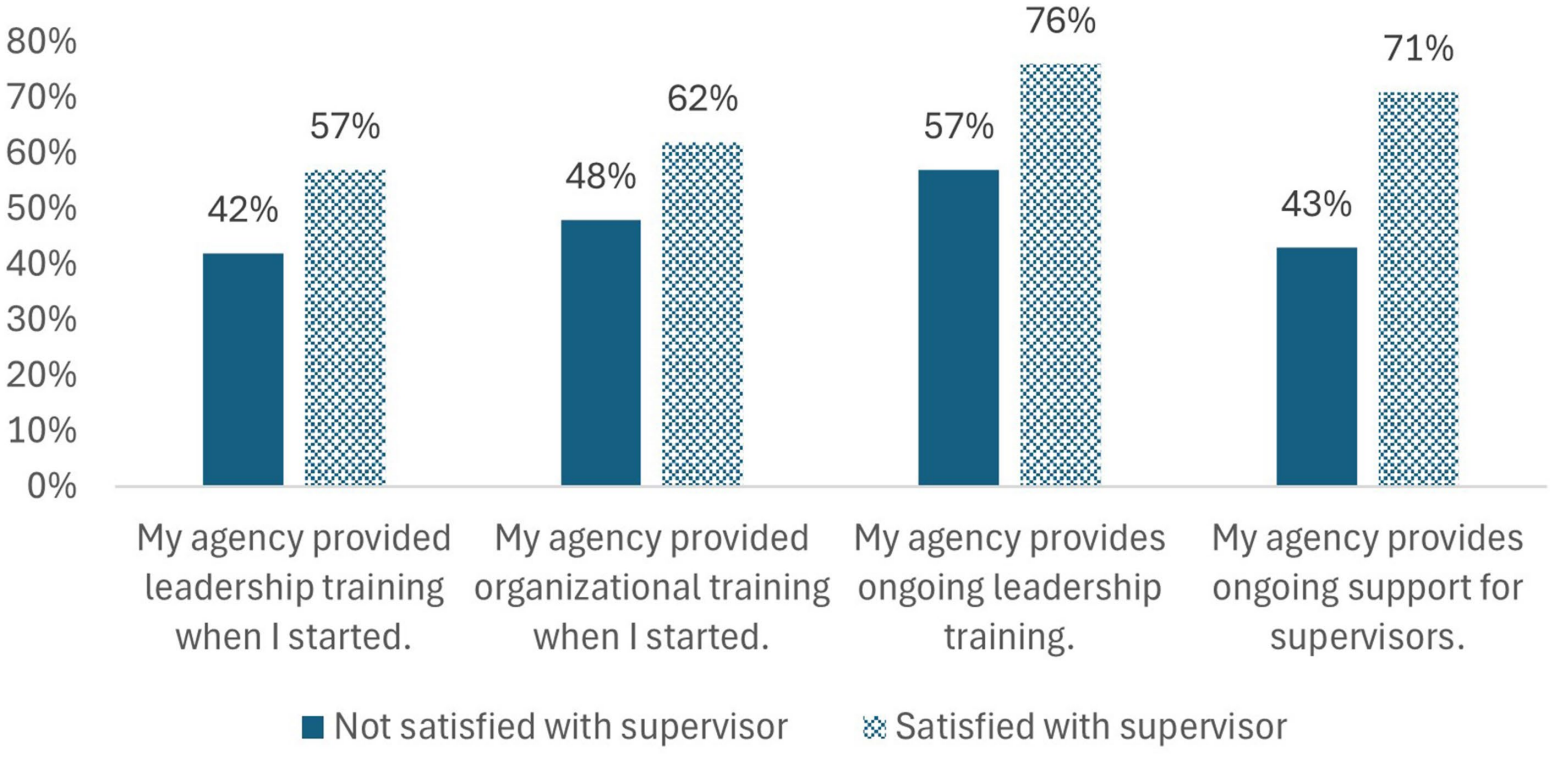
Training and support compared to non-supervisors’ satisfaction with their supervisors.

In the multi-level analysis at the agency level (Figure 1), a higher proportion of non-supervisors who reported satisfaction with their supervisors had supervisors who were provided with initial leadership training (57% vs. 42%), initial organizational training (62% vs. 48%), ongoing leadership training (76% vs. 57%), and ongoing support (71% vs. 43%) compared to those who were not satisfied with their supervisor. In the inferential analysis of supervisor satisfaction (Table 4), several demographic characteristics were associated with lower supervisor satisfaction, including not having a male gender identity (*p* < 0.001), and not being a white or Asian staff member (*p* = 0.001). Respondents had higher frequency of reporting supervisor satisfaction where 90–100% of supervisors said supervisor training was provided when starting their position (AOR 1.18, *p* = 0.003), where 75% + of supervisors’ organizations provide ongoing leadership training (AOR 1.12, *p* = 0.036 for 75–90% and AOR 1.09, *p* = 0.096 for 90–100%). Additionally, ongoing support appears highly associated with supervisor satisfaction, all else equal. Compared to staff in programs and agencies where <50% of supervisors report ongoing support, higher mean supervisor support was associated with higher frequency of satisfaction (AOR ranges 1.14–1.38 by group, all *p* < 0.005). Values differed slightly across program areas.

**Table 4:** Correlates of supervisor satisfaction in a multi-level analysis.

		Adjusted OR	95% CI	*p*-value
My agency provided leadership training when I started^*^	<50%	(ref)		
	50–74.9%	1.02	[0.94–1.1]	0.660
	75–89.9%	1.04	[0.93–1.17]	0.472
	90–100%	1.01	[0.9–1.13]	0.885
My agency provided organizational training when I started^*^	<50%	(ref)		
	50–74.9%	1.03	[0.95–1.12]	0.461
	75–89.9%	1.03	[0.92–1.15]	0.610
	90–100%	1.18	[1.06–1.32]	0.003
My agency provides ongoing leadership training^*^	<50%	(ref)		
	50–74.9%	0.98	[0.89–1.08]	0.706
	75–89.9%	1.12	[1.01–1.25]	0.036
	90–100%	1.09	[0.99–1.2]	0.094
My agency provides ongoing support for supervisors^*^	<50%	(ref)		
	50–74.9%	1.14	[1.05–1.25]	0.003
	75–89.9%	1.17	[1.05–1.3]	0.005
	90–100%	1.38	[1.24–1.52]	<0.001
Age	≤35	1.09	[1.01–1.18]	0.022
	36–49	0.99	[0.92–1.06]	0.706
	50–64	(ref)		
	65+	1.15	[0.99–1.32]	0.059
Gender	Male	(ref)		
	Female	0.82	[0.76–0.88]	<0.001
	All Other	0.54	[0.43–0.68]	<0.001
Race & Ethnicity	American Indian or Alaska Native	0.64	[0.44–0.93]	0.021
	Asian	1.03	[0.92–1.17]	0.586
	Black or African American	0.85	[0.78–0.92]	<0.001
	Hispanic or Latino	0.87	[0.8–0.95]	0.002
	Middle Eastern or North African	0.71	[0.48–1.04]	0.081
	Native Hawaiian or Pacific Islander	0.41	[0.28–0.59]	<0.001
	White	(ref)		
	Multiracial and/or Multiethnic	0.70	[0.61–0.81]	<0.001
Setting	SHA-CO	(ref)		
	Large LHD	0.87	[0.82–0.94]	<0.001
	Medium LHD	1.06	[0.96–1.17]	0.227
	Small LHD	1.21	[0.96–1.51]	0.103
Supervisory Status	Non-supervisors	(ref)		
	Supervisors & Managers	0.96	[0.9–1.02]	0.216
	Executives	1.24	[1.04–1.48]	0.016
Primary Program Area	Assessment & Surveillance	0.73	[0.66–0.8]	<0.001
	Chronic Disease & Injury Prevention	0.91	[0.79–1.06]	0.230
	Clinical Health Care & Social Services	1.00	[0.91–1.11]	0.949
	Communicable Disease Control	0.85	[0.76–0.95]	0.005
	Communications & Policy	0.79	[0.68–0.91]	0.002
	Emergency Preparedness & Response	0.71	[0.58–0.85]	<0.001
	Environmental Public Health	0.88	[0.78–0.99]	0.027
	Maternal, Child, & Family Health	0.96	[0.87–1.06]	0.398
	Organizational Competencies	(ref)		
	Other	0.94	[0.78–1.12]	0.472
	Intercept	7.78	[6.82–8.87]	<0.001

This analysis does not employ survey weights, owing to intra-agency analysis conducted.

* Calculated as mean percent [variable] agree/strongly agree among supervisors, managers, and executives by agency and program area. Instances where supervisors or higher were not present in an agency/program area were excluded from analysis.

## 4 Discussion

The government public health workforce continues to face mounting challenges — from constrained resources and workforce attrition to increasing politicization and public distrust. While many of these systemic issues require long-term structural solutions, this study identifies a critical and addressable gap that has immediate implications for workforce development and retention: the lack of consistent, structured leadership training and support for supervisors.

Supervisors serve as a crucial link between leadership and staff, directly shaping employee experiences, organizational culture, and retention (7–9). Yet findings from this study underscore a widespread absence of institutional investment in supervisory development. Nearly half of supervisors reported receiving no leadership training (46%) or organizational training (41%) when entering their current supervisory role. Furthermore, a substantial proportion reported no ongoing leadership training (27%) or support (31%). These gaps are particularly concerning given that 38% of supervisors have less than 5 years of public health management experience, and nearly half (46%) have five or fewer years of supervisory experience prior to their current position, indicating a relatively inexperienced leadership cohort with high developmental needs.

The consequences of this underinvestment are evident in workforce perceptions. Among non-supervisors, 14% expressed dissatisfaction with their supervisor and 18% reported that their supervisor lacked effective people management skills. Importantly, dissatisfaction with supervision was cited by 23% of non-supervisors intending to leave their jobs in the next year, highlighting the tangible link between supervisory quality and employee retention. However, the data also point to a clear and actionable solution. Multilevel analyses revealed that non-supervisors who were satisfied with their supervisors were significantly more likely to work in agencies where supervisors had received leadership and organizational training — both at the onset of their roles and through ongoing development. Specifically, satisfaction was higher in agencies where supervisors received initial leadership training, ongoing leadership training, and ongoing support.

These findings suggest that training and support for supervisors are not merely procedural or ancillary — they are essential levers for cultivating strong leadership, improving workplace culture, and strengthening organizational resilience. Agencies that consistently invest in these systems produce supervisors who are more effective, more responsive to their teams, and more likely to retain staff. This holds significant implications for workforce strategy, particularly given the cost and disruption associated with turnover in the public health sector.

Investments in leadership development are standard practice in the private sector, where leadership training is widely recognized as a key business priority. According to the Association for Talent Development (13), U.S. companies spent an average of $1,300 per employee on direct learning expenditures in 2023, with leadership development consistently ranking as one of the top areas of investment. Research demonstrates that organizations with strong leadership development programs are more likely to be high performers with better employee engagement, lower turnover, and improved organizational outcomes (14, 15). Leadership development has been shown to produce a positive return on investment by reducing attrition, increasing productivity, and enhancing organizational agility — outcomes that are especially critical in the public health sector. Adapting effective leadership development from the private sector to state and local public health agencies, alongside securing sustained funding for the programs, is key to strengthening the public health workforce.

Leadership development must be understood as a continuous process. Onboarding is essential to equip new supervisors with basic competencies in communication, conflict resolution, policy navigation, and personnel management. Equally important is ongoing support, such as coaching, peer learning networks, and advanced training tailored to evolving workforce challenges (3, 4, 6). Without these systems in place, supervisors are left to navigate complex roles without the necessary preparation or support, to the detriment of both staff and agency performance.

### 4.1 Limitations

This study has several limitations. First, supervisors self-reported their experiences with agency training and support, which may not accurately reflect the actual practices within their agencies. Second, non-supervisors could not be directly linked to their specific supervisors; instead, the analysis relied on multilevel modeling that accounted for agency and program area in which the respondents work. Finally, the cross-sectional design limits the ability to draw causal conclusions, allowing only for the identification of associations.

### 4.2 Conclusion

While many challenges facing the government public health workforce are complex and multifaceted, leadership development is a domain where progress is both feasible and impactful. Strengthening leadership capacity is especially urgent in the current climate, as public health agencies face growing scrutiny and a decline in public trust. Investing in leadership development can help equip agencies with the skills and resilience needed to navigate these pressures. Schools of public health and governmental agencies alike have a shared responsibility to elevate supervisory training as a core element of workforce infrastructure. By embedding leadership development into the lifecycle of supervisory roles — from initial appointment through ongoing tenure — agencies can create environments where leaders thrive, employees feel supported, and the public health system is better equipped to meet current and future challenges. Future research should identify effective leadership development curricula and support strategies that can be readily adapted and implemented across public health agencies. Building better public health leaders in government public health agencies begins with building better systems to train and support them. Agencies that prioritize this work will not only enhance the capacity of their leadership but also contribute to a more stable, satisfied, and resilient public health workforce.

## Data Availability

The raw data supporting the conclusions of this article can be made available upon request. Please visit www.phwins.org to submit a request.
